# Development of machine learning model to predict pulmonary function with low‐dose CT‐derived parameter response mapping in a community‐based chest screening cohort

**DOI:** 10.1002/acm2.14171

**Published:** 2023-10-02

**Authors:** Xiuxiu Zhou, Yu Pu, Di Zhang, Yu Guan, Yang Lu, Weidong Zhang, Chi‐Cheng Fu, Qu Fang, Hanxiao Zhang, Shiyuan Liu, Li Fan

**Affiliations:** ^1^ Department of Radiology Second Affiliated Hospital of PLA Naval Medical University Shanghai China; ^2^ Shanghai Aitrox Technology Corporation Limited Shanghai China

**Keywords:** chronic obstructive, pulmonary disease, pulmonary function test, quantitative imaging, tomography, X‐ray computed

## Abstract

**Purpose:**

To construct and evaluate the performance of a machine learning‐based low dose computed tomography (LDCT)‐derived parametric response mapping (PRM) model for predicting pulmonary function test (PFT) results.

**Materials and methods:**

A total of 615 subjects from a community‐based screening population (40–74 years old) with PFT parameters, including the ratio of the first second forced expiratory volume to forced vital capacity (FEV1/FVC), the percentage of forced expiratory volume in the one second predicted (FEV1%), and registered inspiration‐to‐expiration chest CT scanning were enrolled retrospectively. Subjects were classified into a normal, high risk, and COPD group based on PFT. Data of 72 PRM‐derived quantitative parameters were collected, including volume and volume percentage of emphysema, functional‐small airways disease, and normal lung tissue. A machine‐learning with random forest regression model and a multilayer perceptron (MLP) model were constructed and tested on PFT prediction, which was followed by evaluation of classification performance based on the PFT predictions.

**Results:**

The machine‐learning model based on PRM parameters showed better performance for predicting PFT than MLP, with a coefficient of determination (R^2^) of 0.749 and 0.792 for FEV1/FVC and FEV1%, respectively. The Mean Squared Errors (MSE) for FEV1/FVC and FEV1% are 0.0030 and 0.0097 for the random forest model, respectively. The Root Mean Squared Errors (RMSE) for FEV1/FVC and FEV1% are 0.055 and 0.098, respectively. The sensitivity, specificity, and accuracy for differentiating between the normal group and high‐risk group were 34/40 (85%), 65/72 (90%), and 99/112 (88%), respectively. For differentiating between the non‐COPD group and COPD group, the sensitivity, specificity, and accuracy were 8/9 (89%), 112/112 (100%), 120/121 (99%), respectively.

**Conclusions:**

The machine learning‐based random forest model predicts PFT results in a community screening population based on PRM, and it identifies high risk COPD from normal populations with high sensitivity and reliably predicts of high‐risk COPD.

## 1 INTRODUCTION

In the current aging society, chronic non‐communicable diseases have become a major burden on healthcare. Low dose chest CT screening has been widely promoted in China. Chest CT images not only provides information on pulmonary nodules, but also further evaluates Emphysema, coronary artery calcification, etc. in community population. Occasional lesions in LDCT lung cancer screening are not uncommon, and most of these abnormal findings do not have significant clinical significance and do not require further examination and treatment.^1^ However, there are still some abnormal manifestations indicating that the subjects have potential health hazards, especially some noncommunicable chronic disease (NCD) that are highly prevalent in the aging society, which may be the main reason for the decrease in all cause mortality (ACM) beyond lung cancer. LDCT is superior to other organs in displaying lung diseases and can be used to observe common lung lesions such as chronic obstructive pulmonary disease (COPD). Pulmonary function tests (PFTs) are the detection methods used to diagnose chronic obstructive pulmonary disease (COPD). The diagnostic information provided by PFTs is limited and cannot accurately screen high‐risk COPD patients. In fact, many articles published in the *American Journal of Respiratory and Critical Care*, *Lancet*, and *Nature Outlook* ^2^, ^3^, ^4^ have appealed to physicians to pay more attention to the role of imaging in the early diagnosis of COPD.

Related studies have found that small airway remodeling or vascular remodeling occurs before the pulmonary parenchyma destruction.^5^, ^6^ A certain number of asymptomatic patients in the chest disease screening population will have small airway diseases. Therefore, early diagnosis of small airway abnormalities is very important, particularly since functional small airway disease (fSAD) is reversible. At this stage, however, ability of the lung function tests to detect any early abnormality is limited. Air trapping is the index used most commonly to evaluate small airway disease. However, it is difficult to differentiate the cause of air trapping from emphysema or non‐emphysema fSAD. fSAD is a reversible transitional stage between normal lung tissue and emphysema, which occurs earlier than emphysema. Parametric response mapping (PRM), a recently developed CT quantitative parameter, is based on changes in voxel density between the paired inspiratory and expiratory CT images.^7^ At present, studies about PRM focus mostly on the quantitative evaluation of functional small airway, correlation between PRM and PFT tests, and matching of PRM with functional MR imaging.^8^, ^9^, ^10^, ^11^ Moreover, PRM has demonstrated good sensitivity in the evaluation of disease progression.^12^

Artificial intelligence (AI) has accelerated the progress of COPD research, including in emphysema detection and subtype classification, early screening, and diagnosis.^13^, ^14^ PRM has been shown to correlate positively with PFT parameters.^10^ In recent years, many studies have used PRM to construct predictive COPD/non COPD models,^9^, ^15^ which have achieved good results. However, studies about whether PFT parameters can be predicted based on the PRM from dual phase LDCT have not been retrieved to improve our limited knowledge in the community population. An AI algorithm that predicts PFT parameters based on PRM from LDCT scanning would greatly increase the value of one‐stop CT scanning to extract more vital information about the pulmonary function status.

### 1.1 Related work

Related work can be divided into two categories according to the study purposes and approaches: one focuses on finding the correlation between PRM parameters and PFT parameters from a clinical perspective using traditional statistical tools,^16^, ^17^, ^18^, ^19^ such as the multivariate linear regression model; the other focuses on computer‐aided diagnosis (CAD) of COPD, including patient classification and scoring, based on AI techniques.

In terms of the clinical studies on the PRM‐PFT correlation, Bhatt et al.^10^ constructed multivariate linear regression models and found an association between the PRM parameters and FEV1 annual decline, claiming that the association was of greater importance for patients with mild COPD. Pompe et al.^20^ also used the multivariate linear regression model, finding that PRM^fSAD^ was associated with total lung capacity (TLC), alveolar volume (VA) and residual volume (RV). The regression model in that study had an R^2^ of 0.69 for PRM^fSAD^ prediction. In a similar study, Capaldi et al.^21^ performed multivariate linear regression using a reversed direction to regress PRM parameters with PFT parameters, finding that PRM gas trapping was predicted by FEV1/FVC, and that PRM emphysema was predicted by carbon monoxide diffusion capacity and ventilation defect percentage (VDP).

In terms of the studies on AI‐based CAD for COPD, researchers used AI models for emphysema detection, differential diagnosis, and COPD assessments. Ho et al.^22^ constructed a 3D‐CNN deep learning network to distinguish COPD patients from non‐COPD subjects based on 2D or 3D PRM image input. The accuracy and sensitivity of their classification model was 89.3% and 88.3%, respectively. Besides the attempts to use AI models to distinguish COPD and non‐COPD cases, Humphries et al.^23^ constructed a CT image‐based deep‐learning model for emphysema scoring, which corresponded well with visual scoring (κ = 0.60). Besides, several studies attempted to construct regression models to predict PFT parameters directly from CT images. Li et al.^24^ constructed a 3D deep learning model to predict PFT parameters, with an R^2^ of 0.57 for FEV1 prediction and 0.66 for FEV1% prediction. Schabdach et al.^25^ reported a non‐parametric FEV1 regression method with an R^2^ of 0.55. Singla et al.^26^ proposed a novel method and reported the best regression performance to date, with an R^2^ of 0.68 for FEV1 and 0.71 for FEV1/FVC.

Inspired by these studies, we aimed to construct PRM‐based artificial intelligence algorithms, including a random forest model and a multi‐layer perceptron model, to predict key PFT parameters and to evaluate their performance in a community‐based LDCT screening population undergoing one‐stop screening for Big Three chest diseases (lung cancer, COPD, and cardiovascular disease).

## 2 MATERIALS AND METHODS

### 2.1 Patient population

**FIGURE 1:**
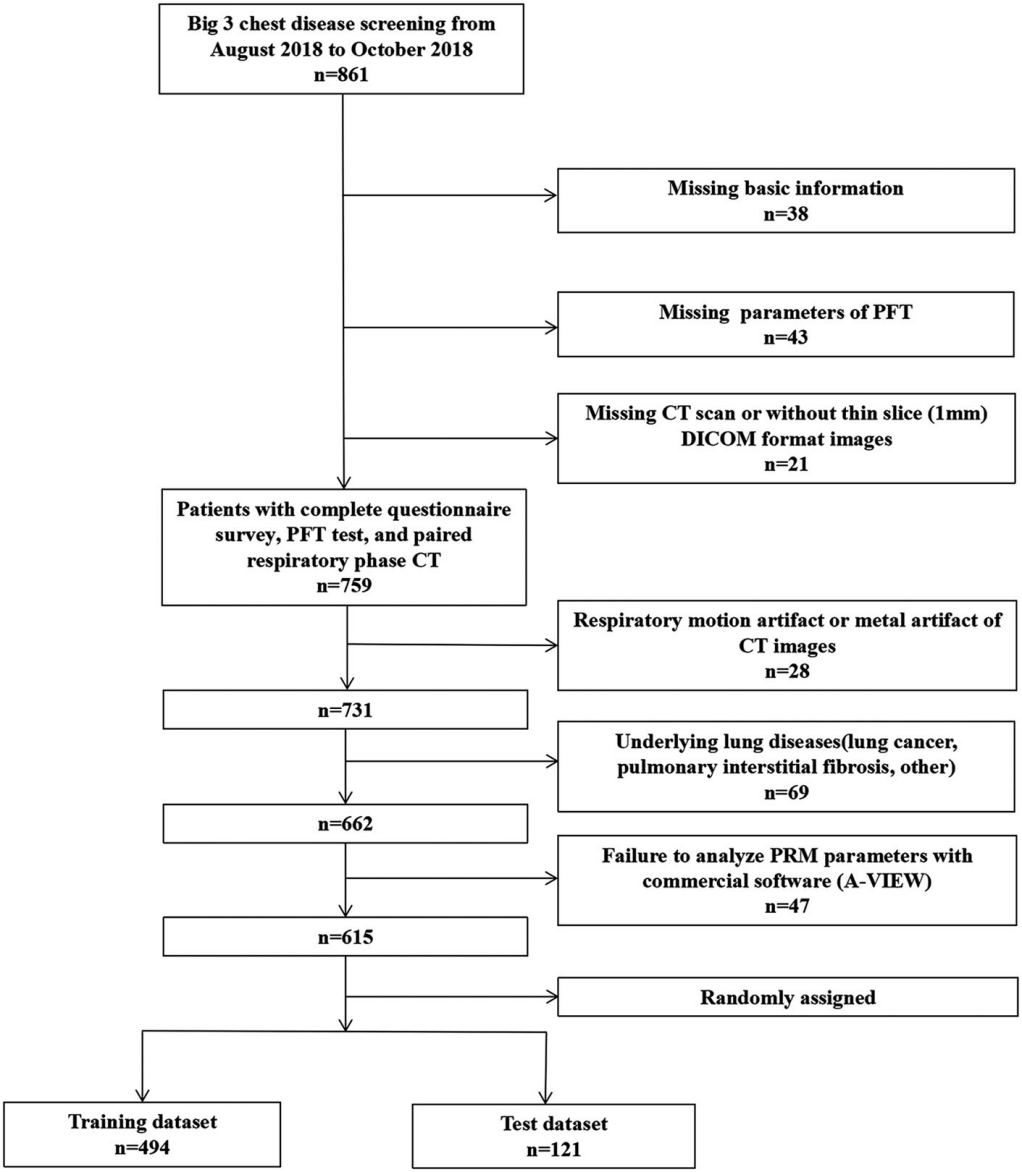
Study selection and baseline characteristics.

From August 2018 to October 2018, a total of 861 consecutive community‐based participants were screened for the Big Three chest diseases in our hospital and the PRM data of 615 participants were collected retrospectively. The patient selection process is shown in Figure 1. Inclusion criteria were as follows: participants with complete questionnaire surveys, PFT test and paired respiratory CT scanning. Exclusion criteria were: (1) marked respiratory motion or metal artifact on CT images; (2) without thin slice (1 mm) DICOM format images; (3) underlying lung diseases such as lung cancer, severe pulmonary interstitial fibrosis, severe pulmonary tuberculosis, asthma, massive pulmonary infection, acute pulmonary embolism, or pulmonary infarction; (4) thoracic deformity; (5) pleural effusion; and (6) chest surgery history. Based on their PFT parameter values, the participants were classified into a normal group, high‐risk group, and COPD group. The normal group was defined as ratio of the first second forced expiratory volume to forced vital capacity (FEV1/FVC) > 0.7 and percentage of forced expiratory volume in the one second predicted (FEV1% predicted value) ≥ 95%. The high‐risk group was defined as FEV1/FVC > 0.7 and 80% ≤ FEV1% predicted value < 95%.^27^ Based on the GOLD criteria, the severity of COPD was classified into GOLD I (FEV1/ FVC < 0.7 and FEV1% predicted≧80%), GOLD II (FEV1/ FVC < 0.7 and 50%≤FEV1% predicted value < 80%), GOLD III (FEV1/FVC < 0.7 and 30% ≤ FEV1% predicted value < 50%), and GOLD IV (FEV1/FVC < 0.7 and FEV1% predicted value <30%). All participants filled out the questionnaire before PFTs, then underwent PFTs and chest CT scanning in the same day.

### 2.2 Pulmonary function tests

PFTs were performed for all patients using the Multifunction Spirometer (HI‐801, CHESTGRAPH, CHEST. MI. Omnia Inc., Japan). PFTs have 15 separate parameters, including FVC, FEV1/FVC, FEV1% and other parameters. FEV1/FVC and FEV1% were the key parameters for analysis in the present study.

### 2.3 CT scanning

All patients underwent breath‐hold training before scanning, taking a supine position with arms above the head. Non‐contrast‐enhanced volumetric chest CT scanning was performed at the end of inspiration and expiration using a 256‐slice CT scanner (Brilliance‐iCT, Philips Healthcare, Cambridge, MA, USA) from the thoracic inlet to diaphragm, respectively. The following CT scanning parameters were used: collimation 128 × 0.625 mm, tube energy 120 kV, Z‐axial and 3D automatic tube current modulation, Dose right on and reduced dose level 3 (inspiratory/expiratory scanning), pitch 0.70, slice thickness 1 mm, slice increment 1 mm, FOV 350 mm × 350 mm, matrix 512 × 512, high and standard resolution algorithms.

### 2.4 PRM analysis

The raw Dicom data of CT images were transferred to the workstation (A‐VIEW, Suhai Information Technology Ltd., Suzhou, China) for PRM analysis. First, a 20‐year experienced thoracic radiologist checked and redefined the lobe segmentation slice‐ by‐slice during the PRM analysis, who was blinded to participants' clinical information and PFT results. Then, the expiratory CT images were registered to the inspiratory CT images at the pixel level. As described previously,^11^ the voxels are divided into four categories according to CT values on paired respiratory CT images: (1) Emphysema, voxels less than or equal to −950HU on the inspiratory image and less than −856HU on the expiratory image; (2) f^SAD^, voxels greater than −950HU on the inspiratory image and less than or equal to −856HU on the expiratory image; (3) Normal lung, voxels greater than −950HU on the inspiratory image and greater than −856HU on the expiratory image; and (4) Uncategorized tissue, voxels less than −950HU on the inspiratory image and greater than −856HU on the expiratory image. The volume as well as the volume percentage of each voxel category (PRM^Emph^, PRM^Emph^%, PRM^fSAD^, PRM^fSAD^%, PRM^Normal^, PRM^Normal^%, PRM^Uncategorized^, and PRM^Uncategorized^%) were calculated at the level of whole lung, left lung, right lung, and each lung lobe, respectively. A total of 72 PRM parameters were measured for each participant.

### 2.5 AI model construction and performance evaluation

Two different types of AI regression models were constructed and trained for the PFT key parameter regression tasks: (1) Random Forest, a machine learning regression model; and (2) Multilayer Perceptron (MLP), which is also known as Artificial Neural Network (ANN). A total of 76 features from each case, including 72 PRM parameters and four clinical features (age, sex, height, and weight), were used as input for our regression models, and the FEV1/FVC or FEV1% were used as the ground truth for regression model training. For each type of AI model, one regression model was established for the FEV1/FVC prediction task, and another regression model was established for the FEV1% prediction task. The dataset was divided into training and validation dataset with the ratio of 4:1 randomly (494 cases for training and 121 cases for validation). The specific method of constructing the models are shown in Supplementary material. Meanwhile, the coefficient of determination (R^2^) was also calculated as a parameter indicating the proportion of variance in the dependent variable that was predictable from the independent variables. The performance of the random forest regression model was evaluated by calculating the Mean Absolute Error (MAE), Mean Squared Error (MSE) and Root Mean Squared Error (RMSE) between the predicted PFT parameters and the PFT measured ground truth. Lower MAE/MSE/RMSE indicates smaller differences between the prediction and ground truth, which means better regression performance. The Spearman correlation was also calculated between the predicted value of the model and the measured PFT value. The parameters FEV1/FVC and FEV1% predicted by the best AI model was further used for the classification tasks.

In the evaluation process of the present study, classification performance on the validation dataset was examined using confusion matrices. Five metrics were calculated, including sensitivity, specificity, positive predictive value (PPV), negative predictive value (NPV), and accuracy.

### 2.6 Statistical analysis

The datasets with normal distribution are expressed as mean ± standard deviation, and the datasets that do not follow normal distribution are presented as median and interquartile range (IQR). Rank sum test or chi square test (SPSS 26.0) was used for age, sex, height, weight and PFT parameters. Other statistical analysis was performed using the R language platform (Version 4.0.0, R Foundation for Statistical Computing, Vienna, Austria). Statistical comparisons between groups were performed using the analysis of variance (ANOVA) test. ANOVA was used for the datasets with normal distribution and equal variance; non‐parametric Kruskal–Wallis Test was used for non‐normally distributed variables. Tukey HSD Test or Nemenyi Test was performed to compare any two groups for datasets with normal distribution and non‐normal distribution, respectively.

## 3 RESULTS

### 3.1 Demographic data, PFT, and PRM parameters of the three groups

Among the 615 participants included in this study, 367 were normal subjects (151 males, 216 females), 194 were high‐risk subjects (102 males, 92 females), and 54 subjects had COPD (36 males and 18 females). No significant differences were found in age (*p* = 0.129 > 0.001) between the three groups. However, significant differences were shown in sex, FEV1/FVC and FEV1% between the three groups (*p* < 0.001) (Table 1).

**TABLE 1:** Demographic data and PFT parameters in normal, high risk, and COPD groups.

	Total (*n* = 615)	Normal group (*n* = 367)	High‐risk COPD group (*n* = 194)	COPD group (*n* = 54)	*p*
Age	68 (65–70)	68 (65–70)	68 (65–71)	67 (65–70)	0.129
Sex					<0.0001
Male	289 (47%)	151 (41%)	91 (47%)	36 (67%)	–
Female	326 (53%)	216 (59%)	103 (53%)	18 (33%)	–
Height(cm)	164 (158–170)	162.98 ± 7.92	165 ± 8.08	166.97 ± 7.22	0.01
Weight(kg)	65 (57.5–72)	64 (56–71)	66 (59.8–75)	67.63 ± 10.96	0.025
FEV1/FVC	0.83 (0.76–0.88)	0.84 (0.80–0.89)	0.83 (0.77–0.88)	0.65 (0.60–0.68)	<0.0001
FEV1%	82.87 (77.52–88.26)	107.32 (101.03–114.90)	85.03 (76.61–90.18)	67.7 ± 21.93	<0.0001

*Note*: Numbers are listed in median (IQR) or mean ± standard deviation.

Abbreviations: COPD, chronic obstructive pulmonary disease; FEV1%, percentage of forced expiratory volume in the one second predicted; FEV1/FVC, ratio of the first second forced expiratory volume to forced vital capacity; PFT, pulmonary function test.

**FIGURE 2:**
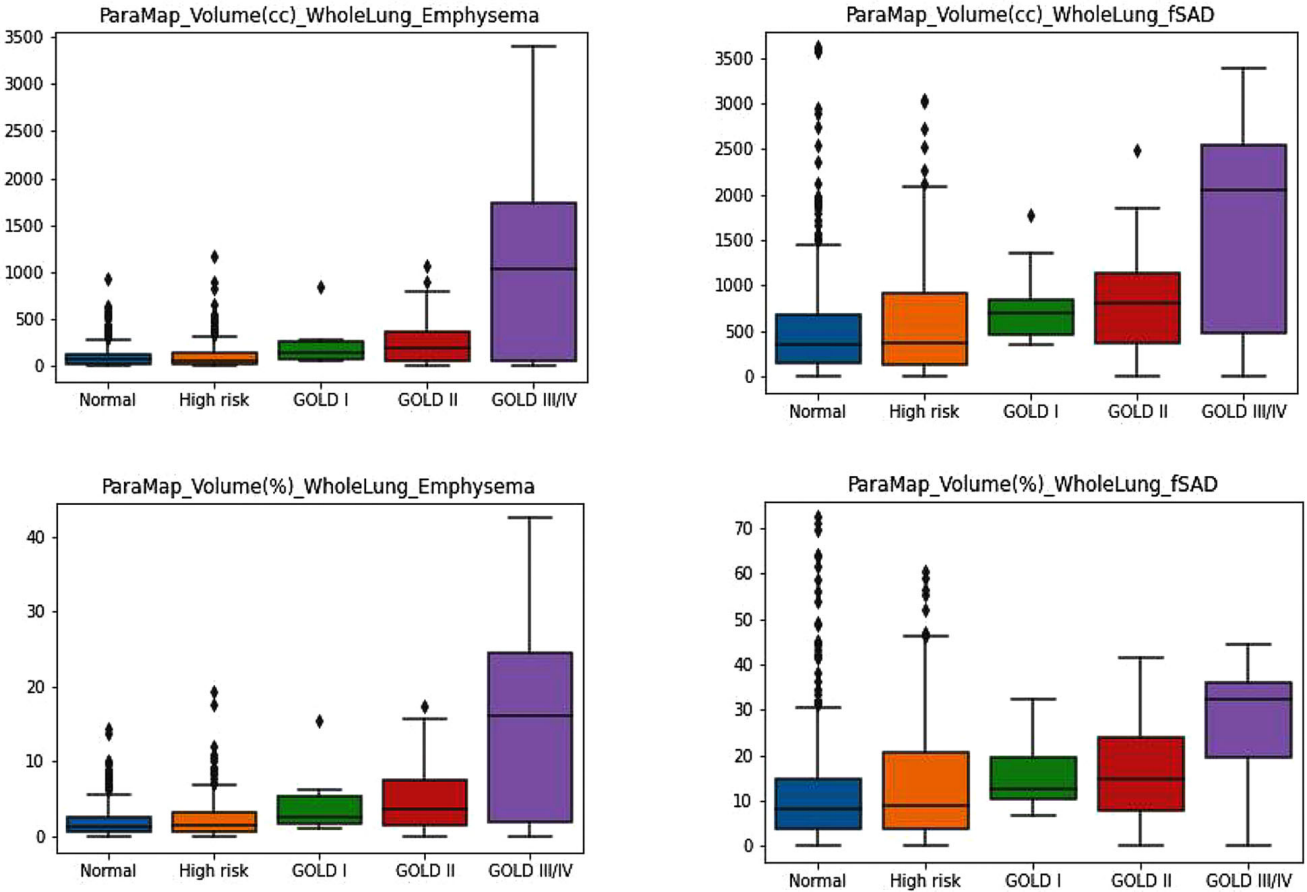
Box plot of PRM^VEmph^, PRM^VEmph^%, PRM^VfSAD^, PRM^VfSAD^% of whole lung.

**FIGURE 3:**
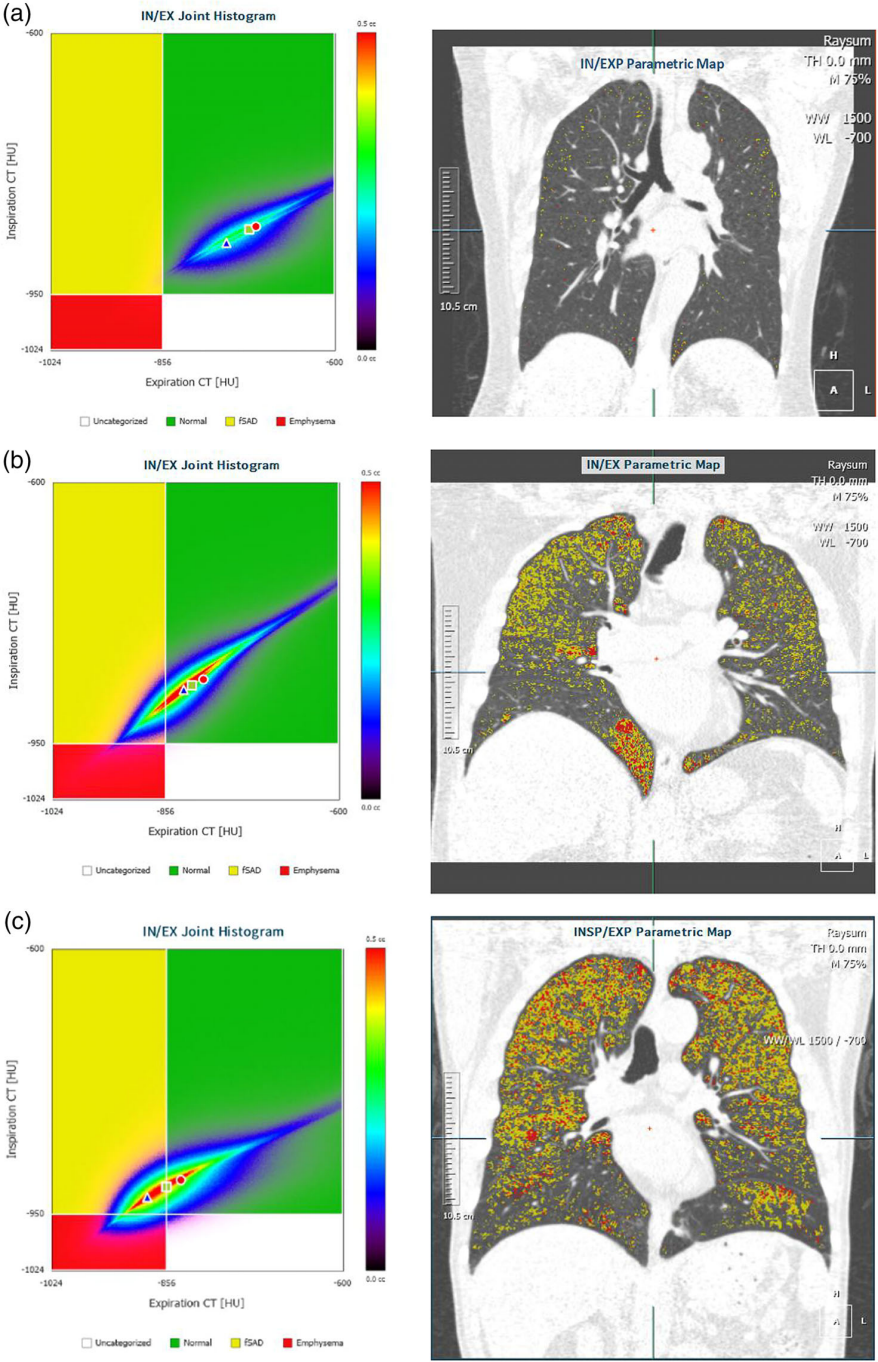
PRM samples of normal, high risk and COPD cases. (a) A normal case, FEV1/FVC = 76.65%, FEV1% = 78.65%. (b) A high risk case, FEV1/FVC = 76.21%, FEV1% = 68.11%. (c) A COPD case, FEV1/FVC = 68.70%, FEV1% = 107.66%.

At the whole lung level, all PRM parameters were different between normal, high‐risk, and COPD groups (*p* < 0.001) (Table 2).The mean values of PRM^V^ ^Emph^, PRM^V^ ^Emph^%, PRM^V^ ^fSAD^, and PRM^V^ ^fSAD^% in the normal group, high risk group and COPD group increased in turn (Figures 2 and 3). Correlations between single PRM and single PFT parameters were very weak (Table S1), as shown in the Supplementary materials.

**TABLE 2:** PRM parameters in normal, high risk, and COPD groups.

PRM parameter	Normal group (*n* = 367)	High‐risk COPD group (*n* = 194)	COPD group (*n* = 54)	*p*
LV	4252.18 (3657.11–5095.83)(a)	4091.36 ± 1079.23(b)	5174.57 ± 1162.40(c)	<0.0001
PRM^VEmph^	62.48 (23.70–128.46)(a)	57.94 (24.17–143.90)(a)	188.25 (65.29–417.28)(b)	<0.0001
PRM^VfSAD^	343.75 (151.68–676.94)(a)	373.16 (128.87–919.86)(a,b)	947.49 ± 756.34(b)	<0.0001
PRM^VNormal^	3661.62 ± 952.56(a)	3302.39 ± 913.54(b)	3695.05 ± 870.24(a,b)	<0.0001
PRM^VUncategorized^	75.12 (31.54–144.70)(a)	55.16 (18.10–107.56)(b)	107.03 (58.85–177.47)(a,b)	<0.0001
PRM^VEmph^%	1.38 (0.62–2.67)(a)	1.48 (0.60–3.20)(a,b)	3.63 (1.61–8.39)(b)	<0.0001
PRM^VfSAD^%	8.17 (3.84–14.81)(a)	8.96 (3.57–20.94)(a)	17.18 ± 10.99(a)	0.001
PRM^VNormal^%	87.72 (80.60–92.35)(a)	86.31 (74.18–93.89)(a,b)	73.70 ± 16.94(b)	<0.0001
PRM^VUncategorized^%	1.78 (0.83–2.91)(a)	1.25 (0.55–2.32)(b)	2.30 (1.20–3.06)(a,b)	<0.0001

*Note*: PRM^VEmph^ and PRM^VEmph^% = the volume of voxels less than or equal to −950HU on the inspiratory image and less than −856HU on the expiratory image of PRM and the volume percentage in whole lung; PRM^VfSAD^ and PRM^VfSAD^% = the volume of voxels greater than −950HU on the inspiratory image and less than or equal to −856HU on the expiratory image and the volume percentage in whole lung; PRM^VNormal^ and PRM^VNormal^% = the volume of voxels greater than −950HU on the inspiratory image and greater than −856HU on the expiratory image and the volume’ percentage in whole lung; PRM^VUncategorized^ and PRM^VUncategorized^% = voxels less than −950HU on the inspiratory image and greater than −856HU on the expiratory image and the volume percentage in whole lung. Numbers are listed in median (IQR) or mean ± standard deviation. The letters a, b and c indicate statistical differences between groups, the letters with repetition indicate no significant statistical differences between groups, and the letters without repetition indicate statistical differences between groups.

Abbreviation: LV, lung volume.

### 3.2 Performance of regression models

For the FEV1/FVC regression task on the test set, the coefficient of determination (R^2^) of the random forest model was 0.749, the MAE was 0.038, the MSE was 0.0030, and the RMSE was 0.055. In contrast, the R^2^, MAE, MSE, and RMSE of the MLP model for the same task are 0.022, 0.063, 0.0069, and 0.083, respectively. For the FEV1% regression task on the test dataset, the R^2^, MAE, MSE, and RMSE for the random forest model were 0.792, 0.068, 0.0097, and 0.098, respectively. Meanwhile, the R^2^, MAE, MSE, and RMSE of the MLP model are −0.109, 0.142, 0.033, and 0.181 for the same task, respectively. The random forest model outperformed the MLP model in both regression tasks, so we used the prediction results of the random forest model for further classifications.

**FIGURE 4:**
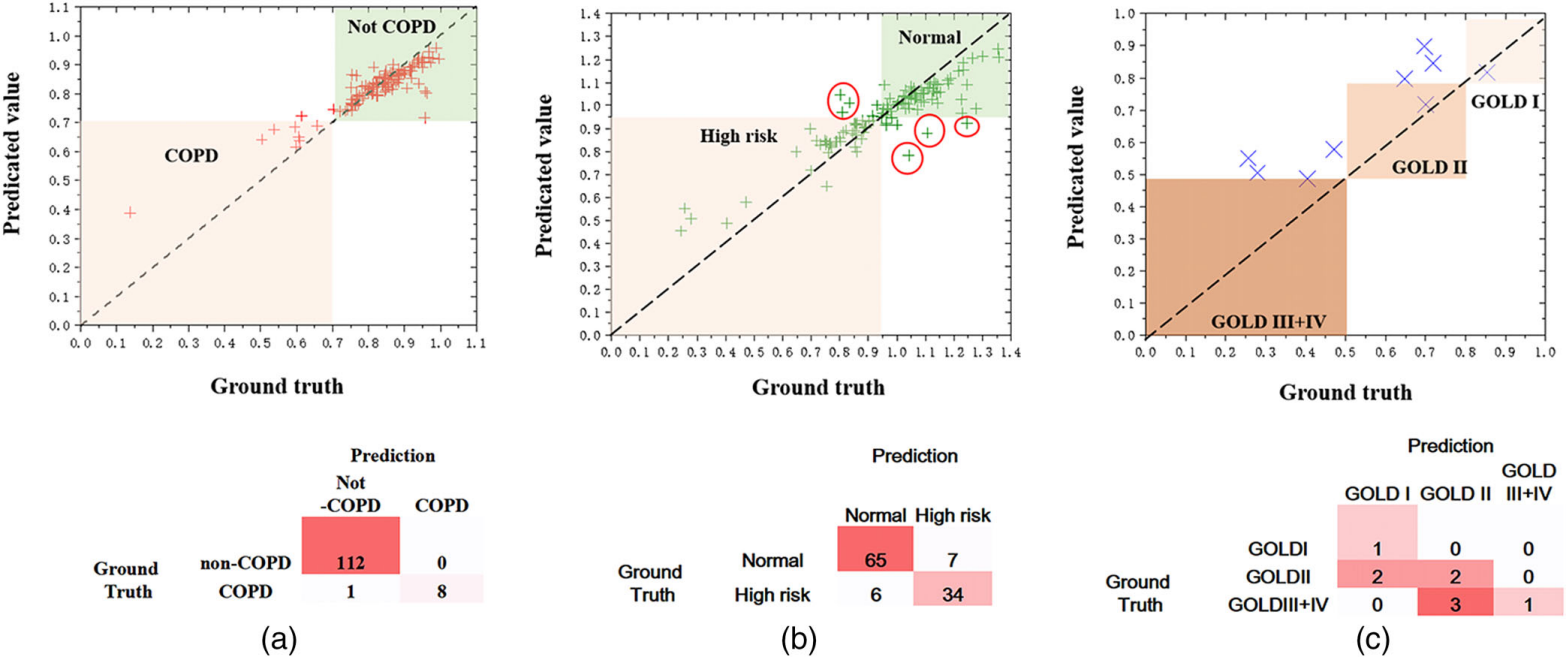
Model performance in the validation dataset. (a) Regression performance for FEV1/FVC prediction in validation dataset and confusion matrix for regression prediction‐ based classification between COPD and non‐COPD group. (b) Regression performance for FEV1% prediction invalidation dataset (for non‐COPD patients) and confusion matrix for the regression prediction‐based classification between normal and high‐risk groups. (c) Regression performance for FEV1% prediction in validation dataset (for COPD patients) and confusion matrix for the regression prediction‐based classification between different GOLD levels.

**FIGURE 5:**
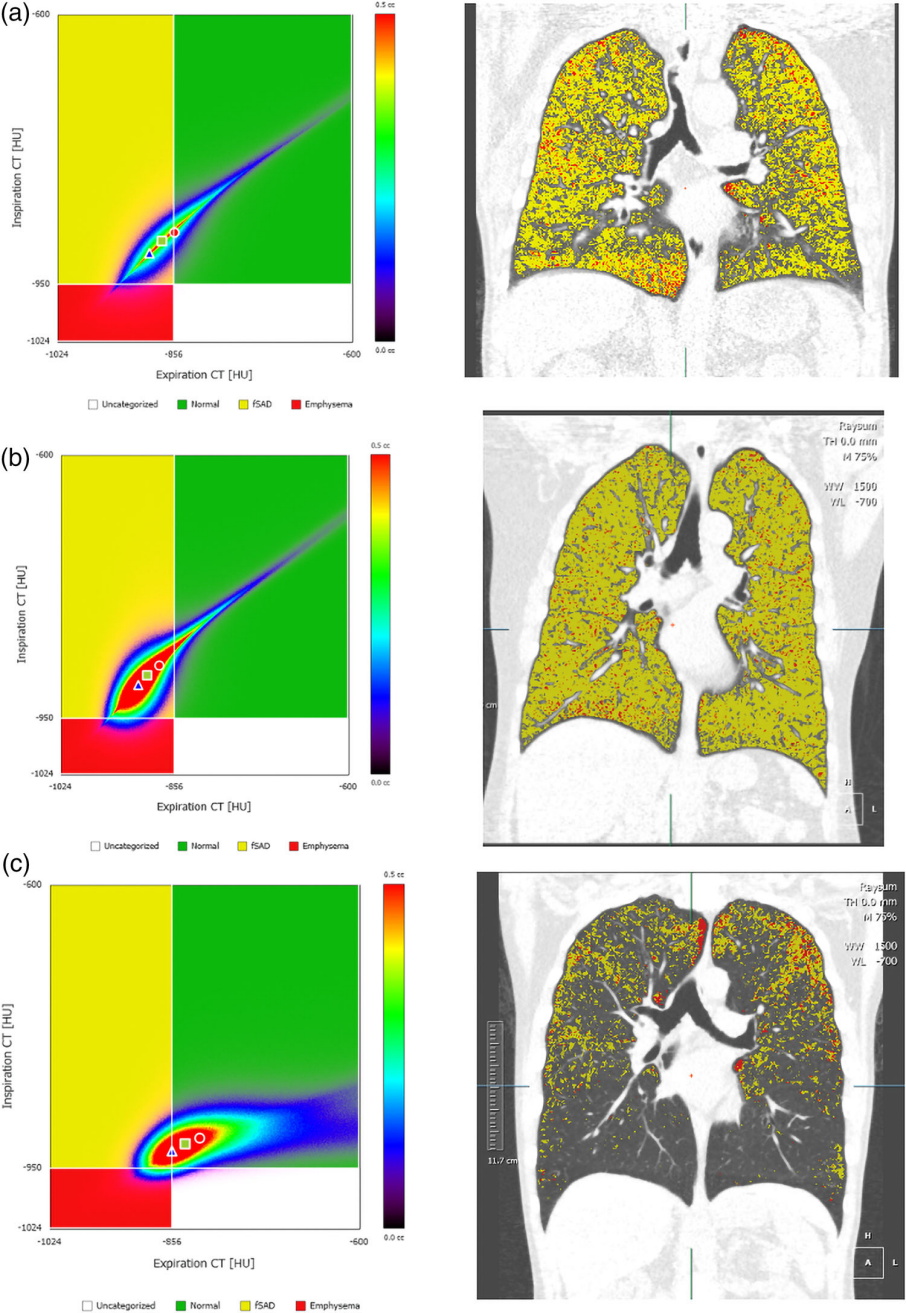
PRM and pulmonary coronal pseudo‐color images of three cases with inconsistent classification results by PFT and prediction model. Three cases with normal PFT were predicted as high‐risk group, which showed greater PRM ^fSAD^%. (a) Male, 66 years old, smoking for 40 years, FEV1/FVC = 0.84, FEV1% = 104.2%. Model prediction results, FEV1% = 78.3%, fSAD% = 19.2%. (b) Female, 69 years old, no smoking history, FEV1/FVC = 0.96, FEV1% = 124.6%. Model prediction results, FEV1% = 92.3%, fSAD% = 69.9%. (c) Male, 44 years old, smoking for 30 years, FEV1/FVC = 0.96, FEV1% = 110.6%. Model prediction results, FEV1% = 87.9%, fSAD% = 13.8%.

For COPD/non‐COPD classification based on the prediction results of the FEV1/FVC regression model, sensitivity was 8/9 (89%), specificity was 112/112 (100%), accuracy was 120/121 (99%), the PPV was 8/8 (100%), and the NPV was 112/113 (99%). For the normal/high risk classification of non‐COPD patients based on the prediction results of the FEV1% regression model, sensitivity was 34/40 (85%), specificity was 65/72 (90%), the PPV was 34/41 (83%), the NPV was 65/71 (92%), and accuracy was 99/112 (88%). In total, 13 participants were in the validation dataset with inconsistent results in classification by PFT and model prediction between normal and high‐risk participants. Among these, significant differences were shown from the ground truth in six participants, as shown in Table 3 and Figure 4 (marked by red circle in the normal group and high‐risk COPD group). Three cases with normal PFT were predicted as the high‐risk group, which showed greater PRM^fSAD^% (Figure 5).

**TABLE 3:** Lung function of cases whose model classification is inconsistent with PFT in normal/high‐risk COPD groups.

	PRM^V^ ^FSAD^%	PRM^VEmph^%	Classification by PFT	FEV1%	FEV1/FVC	FEV1% of model prediction	Classification by model
1	11.3	3.67	High‐risk	0.83	0.94	1.01	Normal
2	22.8	2.37	High‐risk	0.81	0.75	0.97	Normal
3	9.9	0.57	High‐risk	0.8	0.87	1.04	Normal
4	13.8	3.1	Normal	1.11	0.96	0.88	High‐risk
5	69.9	4.85	Normal	1.25	0.96	0.92	High‐risk
6	19.2	2.76	Normal	1.04	0.84	0.78	High‐risk

*Note*: No. = Numbers of 6 cases; PRM^VfSAD^% = the volume percentage of voxels greater than −950HU on the inspiratory image and less than or equal to −856HU on the expiratory image of PRM; PRM^VEmph^% = the volume percentage of voxels less than or equal to −950HU on the inspiratory image and less than −856HU on the expiratory image of PRM.

Abbreviations: COPD, chronic obstructive pulmonary disease; FEV1%, percentage of forced expiratory volume in the one second predicted; FEV1/FVC, ratio of the first second forced expiratory volume to forced vital capacity; PFT, pulmonary function test.

Accuracy was only 44% (4/9) for the GOLD stratification of the COPD patients based on the prediction result of the FEV1% regression model. For all test set data, Spearman correlation was calculated between the predicted value and the measured PFT value. For FEV1/FVC, the Spearman correlation ρ was 0.813 (*p* < 0.001). For FEV1%, the Spearman correlation ρ was 0.846 (*p* < 0.001).

## 4 DISCUSSION

In the present study, AI models, including random forest models and MLP models, were established on the basis of LDCT‐derived PRM parameters to differentiate the normal population from the high‐risk population, and to differentiate the COPD population from the non‐COPD population. These machine‐learning‐based models enhance the clinical value of one‐stop chest CT scanning by predicting PFT results according to PRM parameters.

Functional small airway disease may eventually develop into chronic diseases such as COPD or asthma.^28^, ^29^ PRM is a good predictor for the fSAD. As stated in our literature review, some studies^16^, ^17^, ^18^, ^19^ have focused on the correlation between PRM parameters and PFT parameters using traditional statistical tools. In our study, the correlation between PRM parameters and PFT parameters was shown to be relatively weak, which was similar to the weak PRM‐PFT correlation in the non‐COPD group reported by Capaldi et al.^21^ Considering that non‐COPD patients outnumbered COPD patients (*n* = 561 vs. *n* = 54) in our experiment, the observation was in accordance with the literature. We found that the correlation between PFT and PRM parameters was weak in screening subjects, so random forest and MLP regression models were constructed to regress key PFT parameters. The results showed that the random forest regression model has a much higher performance than the MLP model, which might be due to the lower demand to the quantity and quality of training data set of random forest model than that of MLP.

Table 3 shows the inconsistent classification by PFT and model prediction results between normal and high‐risk COPD groups. High‐risk COPD predicted by our random forest model showed higher PRM^VfSAD^% and PRM^V^ ^Emph^%, suggesting that the model captured functional information such as functional small airway sensitivity, while PRM could not be detected by PFTs. The model was also reliable in distinguishing normal from high‐risk COPD groups. Previous study^30^ also demonstrated that PRM^Emph^% cannot capture the information of GOLD II ∼ IV, but it can distinguish between normal and mild COPD. In our study, PRM parameters were used to predict PFT, which further revealed the clinical potential of PRM for the early management of COPD.

AI has been used to detect emphysema, for differential diagnosis, and to assess the severity of COPD. In our study, the COPD/non‐COPD classification accuracy (99%) and sensitivity (89%) are higher than that published by Ho et al.,^22^ which are 89.3% and 88.3%, respectively. The better classification performance of the present study can be explained by the explicit integration of the PFT parameter‐based COPD and high‐risk definitions in the classification process, while, in contrast, the deep‐learning based classification model implicitly learned it in the training process, which has a high demand on the quality and quantity of image data. We used a simple but practical machine learning method to exploit the clinical value of PRM in community screening of high‐risk COPD patients. Our results indicated a promising potential of this method in clinical practice.

Compared with the studies attempted to construct regression models to predict PFT parameters directly from CT images, the R^2^ values in our study were 0.749 and 0.792 for the FEV1/FVC and FEV1% random forest regression models, respectively, which outperformed previous studies that used deep‐learning approaches. Most deep‐learning approaches used CT images as input, which contained very high dimensional information about the spatial heterogeneity of the lung. In contrast, our feature‐based AI approaches, including random forest models and MLP models, use information extracted from pre‐processed CT images. This may represent an advantage for solving complex issues such as classification of COPD and non‐COPD cases. However, at the same time, the redundant information may also interfere model performance when dealing with other situations such as PFT parameter regression, which was a probable cause for the relatively better result in the present study.

The main limitations of the present study include that it was a single‐center retrospective study without external validation, which limits the extent to which results can be generalized to other populations and cannot rule out selection bias. Multi‐center prospective research should be performed in the future to validate generalization of results. High‐risk COPD was based on one of the published results. As already well known, the criteria of high‐risk COPD are controversial. Therefore, the performance of the machine‐learning model to differentiate normal from high risk may be affected due to the selection of high‐risk criteria. Only PFT results were included for classification, other common clinical history, such as smoking history, was not considered in this study, which may affect the classification of groups to some degree. This study compared machine learning models and artificial neural network algorithm, but did not compare them with deep learning algorithms that use images as inputs. In future work, we need to add other models to further validate the effectiveness of PRM derived from low‐dose chest CT in predicting lung function. All the above factors must be considered as we draw a more accurate and powerful model to predict PFT with PRM from chest CT.

In conclusion, machine‐learning‐based regression models using LDCT‐derived PRM parameters demonstrate good performance in predicting reliable PFT results and classifying normal/high risk patients as well as COPD/non‐COPD patients. More functional information can be captured by this model than by pulmonary function tests, and the prediction results are complementary to PFT under the current evaluation criteria of pulmonary function. Results of the model play a warning role in evaluating the screening population for COPD, which greatly improves the cost‐effectiveness of LDCT.

## AUTHOR CONTRIBUTIONS

Xiuxiu Zhou: Substantial contributions to the conception and design of the work; the acquisition, analysis, interpretation of data for the work. Yu Pu: Design of the work; the acquisition, analysis, interpretation of data for the work. Di Zhang: The acquisition, analysis, interpretation of data for the work. Yu Guan: Drafting the work and revising it critically for important intellectual content. Yang Lu: The acquisition, analysis of data for the work, revising it critically for important intellectual content. Weidong Zhang: The analysis of data for the work. Chi‐Cheng Fu: The analysis of data for the work, revising it critically for important intellectual content. Qu Fang: Substantial contributions to the conception of the work. Hanxiao Zhang: The analysis of data for the work. Shiyuan Liu: The analysis of data for the work, final approval of the version to be published, agreement to be accountable for all aspects of the work in ensuring that questions related to the accuracy or integrity of any part of the work are appropriately investigated and resolved. Li Fan: Substantial contributions to the design of the work, revised the manuscript critically for important intellectual content, final approval of the version to be published, agreement to be accountable for all aspects of the work in ensuring that questions related to the accuracy or integrity of any part of the work are appropriately investigated and resolved.

## CONFLICT OF INTEREST STATEMENT

All authors have no conflicts of interest to declare.

### ETHICAL STATEMENT

This study was approved by the hospital ethics committee and the written informed consent from all subjects were obtained in this study. Clinical Trials Registry Number is ChiCTR2000035283.

## Supporting information

Supporting Information

## Data Availability

The data that support the findings of this study are available on request from the corresponding author. The data are not publicly available due to privacy or ethical restrictions.
