# Supplementary material for: Development of machine learning model to predict pulmonary function with low‐dose CT‐derived parameter response mapping in a community‐based chest screening cohort
Source: J Appl Clin Med Phys. 2023 Oct 2;24(11):e14171. doi: 10.1002/acm2.14171 (PMC10647993; doi:10.1002/acm2.14171)
Supplement: Supplementary file 1 — Supporting Information [file ACM2-24-e14171-s001.docx]

**Supplementary Table 1** Correlations of PFT and PRM parameters by Spearman’s rank correlation analysis.

| PRM | FEV1/FVC | | FEV1% | |
| --- | --- | --- | --- | --- |
|  | r | p | r | p |
| LV | -0.21 | <0.001 | 0.02 | 0.65 |
| PRM^VEmph^ | -0.34 | <0.001 | -0.09 | 0.02 |
| PRM^VfSAD^ | -0.29 | <0.001 | -0.12 | <0.001 |
| PRM^VNormal^ | -0.03 | 0.4 | 0.18 | <0.001 |
| PRM^VUncategorized^ | -0.16 | <0.001 | 0.09 | 0.02 |
| PRM^VEmph^% | -0.34 | <0.001 | -0.12 | <0.001 |
| PRM^VfSAD^% | -0.26 | <0.001 | -0.14 | <0.001 |
| PRM^VNormal^% | 0.31 | <0.001 | 0.14 | <0.001 |
| PRM^VUncategorized^% | -0.13 | <0.001 | 0.14 | 0.01 |

Note:PRM=Parametric Response Mapping; LV=lung volume; PRM^VEmph^ and PRM^VEmph^%=the volume of voxels less than or equal to -950HU on the inspiratory image and less than -856HU on the expiratory image of PRM and the volume percentage in whole lung; PRM^VfSAD^ and PRM^VfSAD^%= the volume of voxels greater than -950HU on the inspiratory image and less than or equal to -856HU on the expiratory image and the volume percentage in whole lung; PRM^VNormal^ and PRM^VNormal^%= the volume of voxels greater than -950HU on the inspiratory image and greater than -856HU on the expiratory image and the volume’ percentage in whole lung; PRM^VUncategorized^ and PRM^VUncategorized^%=voxels less than -950HU on the inspiratory image and greater than -856HU on the expiratory image and the volume percentage in whole lung; FEV1/FVC: Ratio of the first second forced expiratory volume to forced vital capacity; FEV1%: Percentage of forced expiratory volume in the one second predicted.
